# Effects of zinc supplementation on milk production performance and growth status of Bactrian camels

**DOI:** 10.3389/fvets.2025.1677915

**Published:** 2025-11-13

**Authors:** Xinrui Zhou, Menglei Jiang, Muhammad Amjad, Khosbileg, Miaoyu Liu, Zihui Wu, Jiarui Cheng, Jiabin Tang, Linghua Xu, Xuedan Zhu, Liguo Yang, Guohua Hua

**Affiliations:** 1 Key Laboratory of Agricultural Animal Genetics, Breeding and Reproduction of Ministry of Education, College of Animal Science and Technology, Huazhong Agricultural University, Wuhan, China; 2 National Center for International Research on Animal Genetics, Breeding and Reproduction, Frontiers Science Center for Animal Breeding and Sustainable Production, Huazhong Agricultural University, Wuhan, China; 3 Orgoch Camel Milk Industrial Base, Bayannur City, Inner Mongolia, China; 4 Hubei Hongshan Laboratory, Huazhong Agricultural University, Wuhan, China

**Keywords:** Bactrian camels, zinc, lactation performance, calf growth, weight gain

## Abstract

**Introduction:**

Bactrian camels have diverse agricultural roles, yet their nutritional needs, particularly for trace minerals like zinc, are not well understood.

**Methods:**

This study investigated zinc supplementation’s effects on milk production, hump morphology, and hair development in lactating camels, and its impact on growth and hair development in calves. Seventy-nine lactating camels and their calves were assigned to four groups: a control group without zinc and three treatment groups receiving differential zinc sulfate concentrations.

**Results:**

Results indicated that zinc supplementation had no significant effect on milk yield or composition compared to the control. Furthermore, zinc did not improve the proportion of camels with tilted humps across groups. However, Dose 3 (2.00 g/camel/day) significantly stimulated hair growth compared to the control, whereas Dose 1 (1.00 g/camel/day) and Dose 2 (1.50 g/camel/day) showed no significant differences. In calves, zinc demonstrated more pronounced effects: both Dose 1 (0.50 g/camel/day) and Dose 2 (0.75 g/camel/day) doses markedly enhanced body weight gain and hair development compared to the control. Dose 3 did not benefit calves.

**Discussion:**

Collectively, in the present study, zinc at 2.00 g/camel/day improved hair growth in lactating camels without affecting milk production, while 0.50–0.75 g/camel/day enhances growth and hair development in calves, establishing a basis for zinc supplementation in camel husbandry.

## 1 Introduction

Camels encompass both the dromedary (*Camelus dromedarius*) and the Bactrian camel (*Camelus bactrianus*) (1); the dromedary is found in the northern part of Africa, the Middle East, part of Asia, and the Indian subcontinent, while the Bactrian camel is found primarily in inner, central and east Asia (2, 3). The global camel population has reached a total of 42.4 million (4). The proportion of Bactrian camels within the global camel population is estimated at approximately 10% (5). Although domestic camel contributed about 0.42% to the total milk yield of the world (4), camels are predominantly valued for their extended lactation period when compared to cattle in arid ecosystems, with milk production sustaining significantly longer durations (6). Camel milk boasts high protein content, rich essential fatty acids, and low lactose levels (7, 8); camel meat is characterized by its high protein, low fat, and low cholesterol content (9, 10); while camel hair possesses luxurious texture, natural hues, and superior thermal insulation properties (11). Consequently, camels play a vital role in both local economies and global nutrition.

Most Bactrian camels inhabit arid and semi-arid regions (12). Unfortunately, these areas are deficient in mineral elements, which adversely affects animal production and reproduction (13, 14). Furthermore, the shift in camel husbandry systems from the historically mobile herd systems that provided a highly diverse diet to the predominantly settled, semi-intensive systems of today that resulted in standardized diets (typically alfalfa, occasionally supplemented with barley and concentrates) (15, 16). This dietary shift does not necessarily meet nutritional requirements, including those for trace minerals (17). Additionally, during summer, the hot and arid conditions reduced feed intake, which leads to production and slower growth rates of camels (18, 19). Therefore, mineral supplementation in summer may enhance the productivity and growth of Bactrian camels.

Zinc, as a vital trace element, orchestrates fundamental biological processes including protein biosynthesis, carbohydrate catabolism, and enzymatic regulation (20, 21). Its role extends to supporting somatic development and optimizing lactation efficiency in ruminants (22, 23). Zinc deprivation manifests clinically as growth arrest, anorexia, immunodeficiency, alopecia, and dermatopathies (24, 25). Empirical evidence confirms zinc supplementation enhances growth parameters in rams (26) and calves (27–29). However, the nutritional requirements, especially for trace minerals like zinc, remain poorly established for Bactrian camels.

Considering the positive role of zinc in regulating growth and lactation performance, we hypothesis that zinc supplementation may benefit the milk yield or the growth of calves of camel. In the present study, we evaluated graded zinc concentrations on (1) lactational metrics (milk yield and quality) and hair growth in lactating camels; (2) growth trait and hair growth in calves. The objective was to establish an optimal zinc supplementation protocol for augmenting lactation capacity, growth kinetics, and health for Bactrian camels. This investigation advances camelid nutritional science while informing precision husbandry practices for sustainable desert pastoralism.

## 2 Materials and methods

### 2.1 Experimental animal selection and grouping

The trial was conducted at the Ozhgeqi Camel Milk Industrial Base (Urat Hou Banner, Inner Mongolia), utilizing 158 clinically healthy Gobi red camels (*Camelus ferus*) comprising 4 months postpartum lactating camels (*n* = 79, initial body weight: 468.10 ± 19.11 kg, between their 2nd and 3rd lactations) and 4-month-old calves (*n* = 79, initial body weight: 122.11 ± 11.20 kg) in equivalent physiological states. Employing a single-factor completely randomized design, subjects were stratified into four experimental cohorts. Each treatment group contained 19–21 lactating camels or calves.

### 2.2 Animal housing, feeding, and environmental conditions

**Figure 1:**
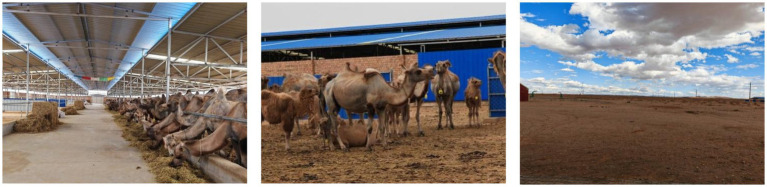
Camel housing, feeding, and environmental conditions.

The trial was conducted during July and August in Bayannur, Inner Mongolia, under hot, arid conditions with sparse grassland coverage (vegetation index ≤30%), Based on the observed physiological manifestations, lactating camels in the region exhibited suboptimal body condition, with 70–80% displaying hump shape deviation (Supplementary Table 1). Subsequent to July onset, these camels demonstrated concurrent manifestations of reduced milk production (Supplementary Table 2) and compromised hair coat quality (neck hair length: 4.37 ± 2.88 cm), while their suckling calves showed growth retardation (body weight: 122.11 ± 11.20 kg) and impaired hair development (neck hair length: 1.79 ± 0.90 cm). All lactating camels and their suckling calves were managed under a semi-extensive management system (Figure 1). Milking is conducted at the facility at 05:00 and 17:00 daily. Prior to milking, suckling calves are guided to the dams to induce suckle-stimulated milk let-down. Following this stimulation, machine milking is initiated. Throughout the experimental period, lactating camels received twice-daily feeding (07:00 and 19:00) of a total mixed ration (TMR) containing 4.5 kg silage, 3.0 kg alfalfa (*Medicago sativa*), and 7.5 kg mixed hay (wheat straw, Mongolian native grass, and rice straw). Suckling calves were provided with 2.0 kg silage, 1.5 kg alfalfa, and 3.5 kg of the same mixed hay formulation. All TMR components were homogenized using a commercial TMR mixer, having *ad libitum* access to water maintained throughout. Additionally, lactating camels received 2.5 kg while their calves received 1.0 kg of concentrate supplement (Table 1) at dusk, formulated as a 1:1 mixture of steam-flaked corn and pelleted supplement.

**Table 1:** Composition of camel essence supplement (%).

Dry matter/%	Crude protein/%	Fat/%	Crude fiber/%	Ca/%	P/%	NaCl/%
89.18	18.88	3.01	9.39	1.09	0.57	1.35

### 2.3 Experimental design and duration

The study lasted for 6 weeks, with an initial 2-week pre-test period. Dietary zinc concentrations were formulated based on NRC (2016) ruminant micronutrient requirements and preliminary dose–response analyses (30). During the testing phase, the zinc sulfate was calculated based on the actual zinc supplementation amounts provided in the group-specific protocols (Table 2), then GB/T 25865-2010-compliant zinc sulfate (Manufacturer: Hebei Yuanda Zhongzheng Biotechnology Co., Ltd., Zinc ≥34.5%) was dissolved in 3 L of potable water per treatment group before administration. The zinc sulfate solution was prepared immediately before using. After being freshly dissolved, the solution was promptly applied to the concentrate supplement using a high-pressure sprayer (25,061, Shanxi xian ABEYINUOR) to ensure complete coverage. To further enhance mixing homogeneity, a semi-closed feed mixing tank was employed, wherein the solution was administered via multiple low-volume sprays over the continuously tumbling feed. This process guaranteed consistent distribution of the supplement. The freshly mixed feed was then promptly provided to the camels to minimize exposure time and ensure consumption while fresh.

**Table 2:** Actual zinc supplementation dose (Zn/day/camel) across control and experimental groups.

Group	Zinc sulfate supplement per camel	
	Lactating camel	Calves
Control	0	0
Dose 1	1.00 g	0.50 g
Dose 2	1.50 g	0.75 g
Dose 3	2.00 g	1.00 g

### 2.4 Milk sample collection and analysis of milk composition

The experiment comprised a pre-test period and a test period. Milk samples were collected before the start of the pre-test period, at the conclusion of the pre-test period, and subsequently, on average, once weekly. Samples were obtained twice daily, once in the morning and once in the evening. Morning and evening milk samples were mixed at a ratio of 1:1 prior to analysis. Then we analyzed milk composition using FOSS milk composition analyzer (MilkoScan FT 6000, manufacturer: FOSS Analytical A/S, country of origin: Denmark,). The analyses were conducted at the DHI Testing Center of the Hubei provincial Livestock and Poultry Breeding Center.

### 2.5 Measurement of body weight in calves

The body weights of calves were recorded using a loadometer (XK3190-A12 + E, Shanghai Yaohua Weighing System Co., Ltd.) before and after the experimental period. Changes in body weight were documented, and bar graphs were generated to illustrate the results. To account for baseline disparities in calf weight that could confound treatment effects, we statistically controlled initial variations using Analysis of Covariance (ANCOVA). This correction was necessary because pre-trial body weight influences post-trial outcomes, and the initial grouping based on lactating camel body condition did not ensure uniform calf body condition across groups, necessitating statistical control for these pre-existing differences. The ANCOVA model adjusted post-trial weights mathematically by fitting a regression line between pre-trial (covariate) and post-trial weights across all groups. Specifically, it calculated adjusted group means (least-squares means) through the formula:

$\begin{array}{c} \text{Adjusted Post}-\text{trial Weight}=\text{Observed Post}-\text{trial Weight}-\beta \\ \;\times (\mathrm{Pre}-\text{trial Weight}-\text{Group Mean}\;\mathrm{Pre}-\text{trial Weight}). \end{array}$

where β is the pooled within-group regression coefficient quantifying the covariate-effect. Zinc supplementation served as the independent variable, post-trial body weight as the dependent variable, and pre-trial body weight as the covariate.

### 2.6 Determination of camel hair length

The length of neck hair on all camels in each group was measured three times during distinct phases of the experiment: early, mid, and late stages. The growth rate of camel hair before and after the experiment was employed as the primary measurement indicator:

$\begin{array}{l} \text{Hair growth rate}=\\ \;\;\;\;\;\;(\text{post}-\text{trial hair length}-\mathrm{pre}-\text{trial hair length})\\ /\text{experiment duration}. \end{array}$

### 2.7 Assessment of lactating camel hump morphology

**Figure 2:**
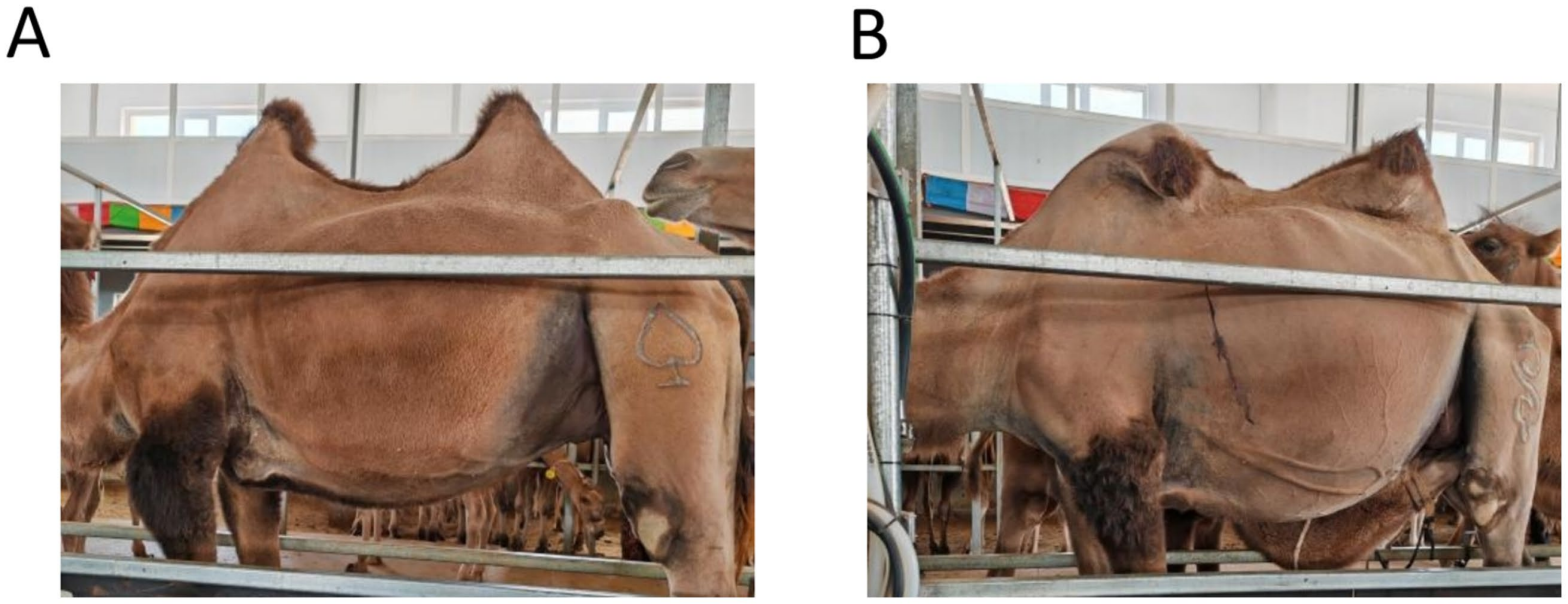
Photo of lactating camel hump morphology. **(A)** full and upright double hump(s), **(B)** tilted single or double hump(s).

Observe and photograph the changes in camel humps before and after the experiment (Figure 2). Categorize the humps into two groups: (Figure 2A) full and upright double hump(s); (Figure 2B) tilted single or double hump(s).

### 2.8 Data analysis and statistics

All data from this experiment were initially analyzed using Excel 2010 and subsequently further processed and visualized using SPSS (version 22.0.0.0; IBM, America) and GraphPad Prism (version 8.0.2; GraphPad Software). Data are reported as mean ± standard error of the mean (Mean ± SEM). One-way analysis of variance (ANOVA) was employed to analyze the effects of zinc supplementation level on milk yield, milk composition, and hair length growth in lactating camels and their calves. Interaction effect analysis was used to examine the interaction between zinc supplementation level and experimental week on milk yield. Bivariate correlation analysis was used to analyze the correlations among milk composition parameters. Analysis of covariance (ANCOVA) was applied to assess the effects of zinc supplementation on the final body weight and daily weight gain of calves at the end of the trial.

## 3 Results

### 3.1 Effects of zinc supplementation on milk production in lactating camels

The effect of zinc supplementation on the milk yield of lactating camels was examined for 6 weeks. During the first month of zinc supplementation, milk yield in all groups had a gradual increasing trend, followed by a slight decline thereafter. However, the results revealed no significant differences (*p* > 0.05) between the treatment groups and controls throughout the experiment (Table 3). Analysis of the interaction between zinc supplementation level and experimental week in Supplementary Table 2 revealed no significant main effect of zinc supplementation on milk yield in lactating camels (*p* > 0.05), whereas experimental week exerted a significant main effect (*p* < 0.05) with no significant interaction effect (*p* > 0.05).

**Table 3:** Effects of different zinc supplementation in diets on lactating camel milk production.

Group	Control	Dose 1	Dose 2	Dose 3	*p-*value
0 week (kg)	1.46 ± 0.22	1.18 ± 0.12	1.33 ± 0.32	1.40 ± 0.07	0.311
2 weeks (kg)	1.75 ± 0.14	1.62 ± 0.36	1.80 ± 0.37	2.10 ± 0.28	0.198
3 weeks (kg)	2.07 ± 0.24	1.94 ± 0.24	1.94 ± 0.25	2.09 ± 0.52	0.871
4 weeks (kg)	2.12 ± 0.37	2.07 ± 0.43	2.13 ± 0.18	1.94 ± 0.12	0.791
5 weeks (kg)	1.90 ± 0.20	1.62 ± 0.17	1.78 ± 0.19	1.81 ± 0.40	0.500
6 weeks (kg)	1.82 ± 0.15	1.66 ± 0.22	1.84 ± 0.22	1.83 ± 0.18	0.504

Data was subjected to one-way analysis of variance (ANOVA).

### 3.2 Effects of zinc supplementation on milk composition in lactating camels

The effects of varying levels of zinc supplementation on the milk composition of lactating camels were investigated. The results showed the milk fat percentage, protein percentage, lactose percentage, solids non-fat (SNF) percentage, and urea of zinc-supplemented groups showed no statistically significant difference (*p* > 0.05; Table 4).

**Table 4:** Effects of various levels of zinc supplementation in diets on the milk composition of lactating camels.

Group	Control	Dose 1	Dose 2	Dose 3	*p-*value
Fat (%)	3.94 ± 0.21	4.04 ± 0.11	4.03 ± 0.17	4.02 ± 0.21	0.978
Protein (%)	3.55 ± 0.08	3.80 ± 0.06	3.70 ± 0.09	3.71 ± 0.08	0.203
Fat/protein (%)	1.09 ± 0.05	1.06 ± 0.02	1.09 ± 0.04	1.08 ± 0.05	0.968
Lactose (%)	5.54 ± 0.13	5.66 ± 0.07	5.66 ± 0.10	5.56 ± 0.09	0.738
SNF (%)	9.79 ± 0.17	10.11 ± 0.09	10.03 ± 0.19	9.96 ± 0.15	0.512
Urea (mg/mL)	29.32 ± 0.95	36.75 ± 2.93	33.12 ± 3.42	31.15 ± 2.46	0.249

Statistical analysis one-way analysis of variance (ANOVA).

As shown in Table 5, milk fat percentage exhibited a positive correlation with milk protein percentage (*r* = 0.72) but a negative correlation with lactose percentage (*r* = −0.25). Lactose percentage was positively correlated with SNF percentage (*r* = 0.93), whereas negatively correlated with urea (*r* = −0.14). Furthermore, SNF percentage demonstrated a negative correlation with urea (*r* = −0.02).

**Table 5:** Correlation analysis of milk composition.

Items	Fat (%)	Protein (%)	Lactose (%)	SNF (%)	Urea (mg/mL)
Fat (%)	1.00	0.72**	−0.25	0.01	0.34
Protein (%)		1.00	0.03	0.38	0.47
Lactose (%)			1.00	0.93**	−0.14
SNF (%)				1.00	−0.02
Urea (mg/mL)					1.00

Statistical analysis bivariate correlation analysis.

### 3.3 Effect of zinc supplementation on weight gain of calves

The effects of different zinc supplementation levels on the body weight and daily gain of calves were examined, with the initial body weight included as a covariate. As shown in Table 6, compared to the control group, the post-trial weight and daily gain of calves in the Dose 1 and Dose 2 groups were significantly higher (*p* < 0.05). However, no significant differences were observed for the Dose 3 group (*p* > 0.05). The results suggest that an additional daily supplementation of 0.50 g or 0.75 g of zinc sulfate per calf promotes weight gain.

**Table 6:** Effects of different doses of zinc supplemented in diets on body weight and daily gain of calves.

Group	Pre-trial weight (kg)	Post-trial weight (kg)	Daily gain (kg/day)	Adjusted post-trial weight (kg)	*p*-value	Adjusted daily gain (kg/day)	*p*-value
Control	125.83 ± 12.56	138.17 ± 11.36	0.41 ± 0.07	134.60 ± 0.62^a^	<0.0001	0.42 ± 0.02^a^	<0.0001
Dose 1	119.25 ± 5.96	136.33 ± 7.29	0.57 ± 0.06	139.08 ± 0.61^b^		0.57 ± 0.02^b^	
Dose 2	116.00 ± 16.66	132.80 ± 16.13	0.56 ± 0.03	138.67 ± 0.70^b^		0.55 ± 0.02^b^	
Dose 3	127.20 ± 5.26	141.00 ± 5.04	0.45 ± 0.02	136.12 ± 0.69^a^		0.46 ± 0.02^a^	

Data analysis utilized Analysis of Covariance (ANCOVA). In the adjusted post-trial weight and adjusted daily gain values, ^a,b^Means with different superscripts within the same column differ significantly (*p* < 0.05).

### 3.4 Effects of zinc supplementation on neck hair growth

**Figure 3:**
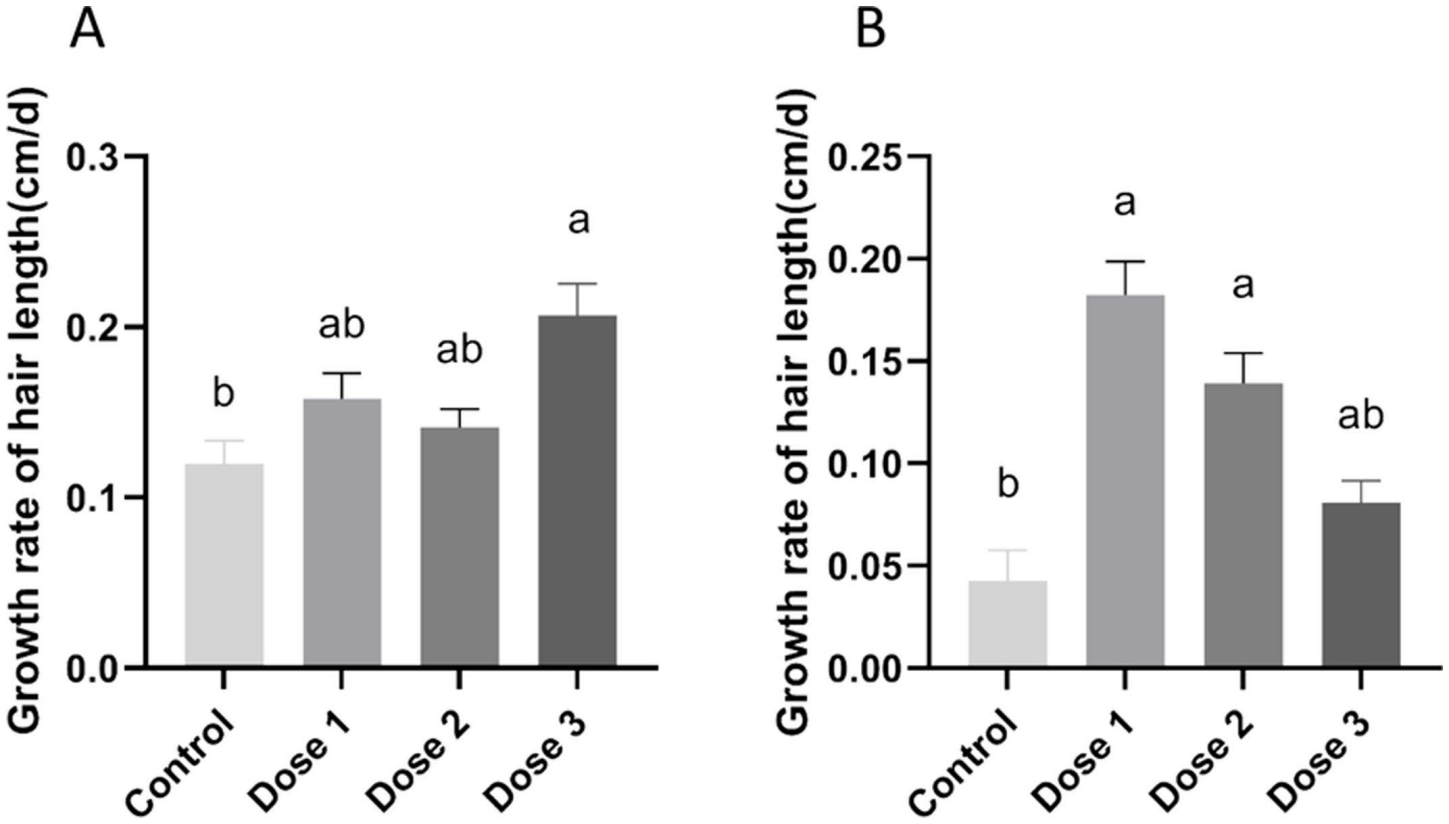
Effect of zinc supplementation on neck hair growth rates. Data analysis utilized one-way ANOVA. **(A)** Growth rate of neck hair in lactating camels. **(B)** Growth rate of neck hair in calves. ^a,b^Different letters indicating significant differences among different groups (*p* < 0.05).

Zinc supplementation significantly influenced the mane growth rates in lactating camels. Specifically, the growth rates of neck hair in the zinc-supplemented groups were significantly faster than those in the control group receiving no zinc supplementation. Notably, the Dose 3 group exhibited a significantly enhanced growth rate compared to the control group (*p* < 0.05), suggesting that varying concentrations of zinc effectively promote hair growth in camels (Figure 3A). The experimental period was divided into the first 2 weeks and the last 2 weeks. Moreover, the results demonstrated the zinc supplementation at Dose 3 superior hair growth promotion in lactating camels during the initial 2 weeks (*p* < 0.05), but no significant differences were observed among the groups in the latter 2-week period (*p* > 0.05; Supplementary Figures 3A,B). Those results show an additional daily supplementation of 2.00 g of zinc per lactating camel significantly stimulate hair growth.

In calves, both the Dose 1 and Dose 2 groups exhibited significantly higher rates of neck hair growth compared to the control group (*p* < 0.05). In contrast, no significant difference was observed between the Dose 3 group and the control group (*p* > 0.05; Figure 3B). As shown in Supplementary Figures 3C,D, zinc supplementation at Dose 1 resulted in the most pronounced improvement in hair growth during the first 2 weeks (*p* < 0.05), whereas doses of 0.50 g and 0.75 g were more effective in the subsequent 2-week period (*p* < 0.05). These results suggest that daily supplementation with 0.50 g or 0.75 g of zinc sulfate per calf effectively promotes hair growth.

### 3.5 Effects of zinc supplementation on hump morphology of lactating camels

Before the trial, the hump morphology of lactating camels in each group was assessed. The hump morphology remained consistent with that observed before the trial after 6 weeks of zinc supplementation (Supplementary Table 1 and Figure 1).

## 4 Discussion

According to the latest data from the FAO, the global camel population consists of approximately 90% dromedary camels. Compared to Bactrian camels, dromedary camel farming is more industrialized, particularly in the Middle East, where they are highly valued for producing camel milk (31, 32), meat, and hair (11, 31, 33). In contrast, this study focuses on exploring the production value of Bactrian camels.

In China, the total population of Bactrian camels is approximately 316,000 (34). They are primarily used for draft labor and hair production. Currently, there is limited and unofficial documented data on the milk yield of Bactrian camels in China. It is reported that an Alxa Bactrian camel can produce 0.25–1.5 kg of milk per day, excluding the amount consumed by the calves (35). Excluding the impact of calves’ consumption, factors such as the quantity and quality of forage, watering frequency, climate, reproductive age, milking frequency, calf care, milking methods, and health status, all affect camel milk production (36). The highest milk yield in Chinese Bactrian camels is observed between the third and fourth months of lactation. However, compared to dromedary and cow milk, Bactrian camel milk particularly that from Chinese Bactrian camels has received less research attention, mainly due to its lower yield (34). Therefore, research and harnessing the production value of Bactrian camels is crucial.

Trace minerals, as essential micronutrients, play crucial roles in regulating numerous biological functions including metabolic processes, production, reproduction, and immunity. In extensive grazing systems, ruminants face high risks of marginal micronutrient deficiencies, leading to reduced livestock production performance and increased susceptibility to various diseases (37–39). This investigation elucidated the impact of varying dietary zinc concentrations on milk production performance in Bactrian camels. Camel milk exhibiting superior mineral content (including calcium, iron, and magnesium) and higher vitamin levels (particularly vitamin A and E) compared to bovine milk (40, 41). Additionally, camel milk has demonstrated immunostimulatory, anticancer, antidiabetic, and antihypertensive properties (42, 43). Given these nutritional advantages and functional benefits, enhancing the camel milk production represents a critical agricultural objective (5). During the 6-week experimental period, milk production (Table 3) and composition (fat percentage, protein percentage, fat to protein ratio, lactose percentage, SNF percentage, and urea) showed no significant differences between experimental and control groups (Table 4). As shown in Table 5, milk fat percentage demonstrated positive correlations with milk protein percentage and fat-to-protein ratio (*r* = 0.715 and 0.972, respectively). Milk protein percentage showed a significant positive correlation with fat-to-protein ratio (*r* = 0.533), while lactose percentage was positively correlated with solids-not-fat (SNF) percentage (*r* = 0.931). The variation in milk composition shows little significant change. Aside from the minimal impact of the zinc supplementation trial, this may also be influenced by mid-infrared spectroscopy technology. The measurement of milk composition using mid-infrared spectroscopy is affected by the animal’s lactation cycle, and the results for various components can vary considerably across different stages of lactation. If more sensitive biosensor technology were employed, it might be possible to detect subtler changes (44). For Maghrebi dairy she-camels, zinc methionine supplementation (Zn-Met) administered from 3 months prepartum until 9 months postpartum significantly improved milk yield and offspring growth performance (13). This result contrasts with our findings, which may be primarily due to interspecies variations—as their study focused on dromedary camels, whereas ours involved Bactrian camels. Additionally, the discrepancy could be explained by the divergent timing of supplementation initiation: their protocol began 3 months prepartum, while our intervention commenced 4 months postpartum. Together, these factors suggest that zinc supplementation at the evaluated concentrations did not significantly enhance milk yield or composition in Bactrian camels.

Milk yield fluctuations across groups may be attributable to environmental variables including temperature variations and grazing conditions (6, 45). Notably, prior to the trial commencement, the local region experienced persistent drought and scarce vegetation. As the experiment progressed, temperatures decreased and precipitation increased, leading to improved climatic conditions. These environmental changes coincided with enhanced milk production. Supplementary Table 2 demonstrated that milk yield in lactating camels increased significantly over the course of the zinc supplementation period. However, this increase was independent of zinc supplementation, indicating that climatic variations likely exerted a substantial influence on lactation performance. Additionally, the milking process at the experimental site requires suckling induction by calves, and the inability to separate nursing calves from lactating dams may influence the total milk yield of the camels (36). Furthermore, the transition from a fully confined system to a semi-grazing management model also impacts lactation performance (44).

The thermoregulatory capacity of camels, facilitated by their dense pelage, enables temperature homeostasis in extreme environments (46). The zinc content in camel fiber was positively correlated with fiber diameter and the proportion of medullated fiber (11). Furthermore, supplemental zinc increased wool fiber growth without increasing fiber diameter (26). Consistent with these findings, this study observed a similar effect. This study focused on neck hair growth measurements due to their accessibility. Zinc supplementation significantly influenced hair growth rates in lactating females and calves. The Dose 3 group had superior growth compared to the control in lactating females (Figure 3A), while the Dose 1 and Dose 2 groups showed significant improvements over untreated animals in calves (Figure 3B). Dietary supplementation with 2.00 g zinc sulfate per camel significantly enhanced hair growth in lactating females, while supplementation with 0.50 g or 0.75 g zinc sulfate per camel significantly promoted hair growth in calves. Otherwise, the optimal zinc supplementation levels for lactating camels and calves were determined as 2.00 g of zinc sulfate per camel and 0.50 g or 0.75 g of zinc sulfate per camel, respectively. Previous studies have reported that the Wnt/*β*-catenin signaling pathway plays a crucial role in hair morphogenesis, growth initiation, and regeneration. We hypothesized that zinc supplementation in the diet of lactating camels and calves may promote fiber growth via the Wnt/β-catenin signaling pathway. This mechanism warrants further investigation (47, 48).

Organ zinc distribution analysis in Mauritanian dromedaries revealed highest muscular zinc concentrations (3.28 mg/100 g), suggesting zinc’s importance in musculoskeletal function (49). Parallel studies in Angus cross calves demonstrated that elevated dietary zinc (particularly as zinc propionate at 1.0 g/head/d) enhanced growth performance without compromising carcass characteristics during β-agonist administration (27, 50). Consistent with the findings, this study demonstrated that daily supplementation with 0.5 or 0.75 g of zinc sulfate significantly enhanced weight gain in 4-month-old calves. Evidence indicates that somatic growth in vertebrates is regulated by the growth hor-mone (GH)/insulin-like growth factor-I (IGF-I) axis (51). Zinc supplementation may potentially stimulate body weight gain in calves through activation of this GH-IGF-I axis.

Camels primarily store energy in the form of fat deposits in their humps and abdominal regions, which maintains their environmental resilience (52, 53). However, this study indicated that local lactating Bactrian camels generally exhibit poor hump morphology, with approximately 80% displaying tilted single or double humps. Notably, 6 months of zinc supplementation failed to restore normal hump morphology in these camels.

As an important agricultural product, camel milk provides significant economic benefits to workers involved in camel husbandry. Apart from its intrinsic nutritional value, discarded or expired milk can now also be repurposed (54). Additionally, the zinc supplementation in this experiment improved coat growth and calf development without affecting milk yield, representing a critical economic aspect for herders.

## 5 Conclusion

In summary, dietary zinc supplementation exerts a positive effect on promoting hair growth in camels and supporting the growth and development of calves. A 6-week zinc supplementation trial revealed no significant improvement in milk yield or milk quality in Bactrian camels. However, daily supplementation of 2.00 g of zinc sulfate per camel significantly promoted neck hair growth while maintaining lactation performance. For calves, daily supplementation of 0.50–0.75 g of zinc sulfate per camel effectively promoted both neck hair development and overall growth. This study provides a scientific basis for formulating targeted zinc supplementation protocols aimed at enhancing camel down production and supporting calf development in arid environments.

## Data Availability

The original contributions presented in the study are included in the article/Supplementary material, further inquiries can be directed to the corresponding author/s.
