# Supplementary material for: Effects of zinc supplementation on milk production performance and growth status of Bactrian camels
Source: Front Vet Sci. 2025 Nov 13;12:1677915. doi: 10.3389/fvets.2025.1677915 (PMC12659195; doi:10.3389/fvets.2025.1677915)
Supplement: Supplementary file 1 [file Data_Sheet_1.docx]

Supplementary Material

**Supplemental Table 1.** The proportion of the two types of hump morphology (A)full and upright double hump(s); (B)tilted single or double hump(s).

| **Group** | **Hump morphology** | |
| --- | --- | --- |
|  | **A** | **B** |
| Control | 27% | 73% |
| Dose 1 | 21% | 79% |
| Dose 2 | 20% | 80% |
| Dose 3 | 26% | 74% |


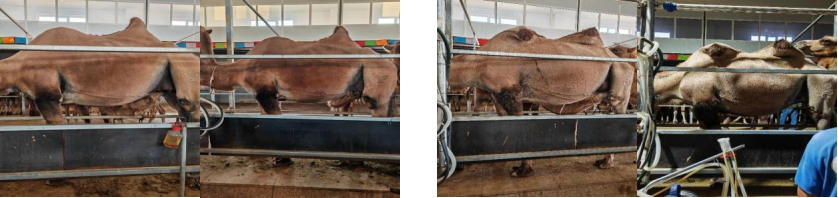

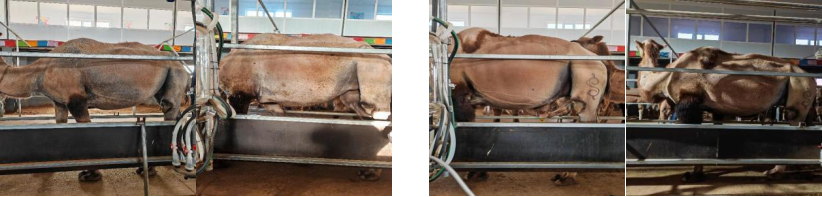


11 (pre-trial)       11 (post-trial)                 10 (pre-trial)        10 (post-trial)

3 (pre-trial)        3 (post-trial)                   111 (pre-trial)          111 (post-trial)

**Supplemental Figure 1**. Photographs of hump morphology before and after the experiment.


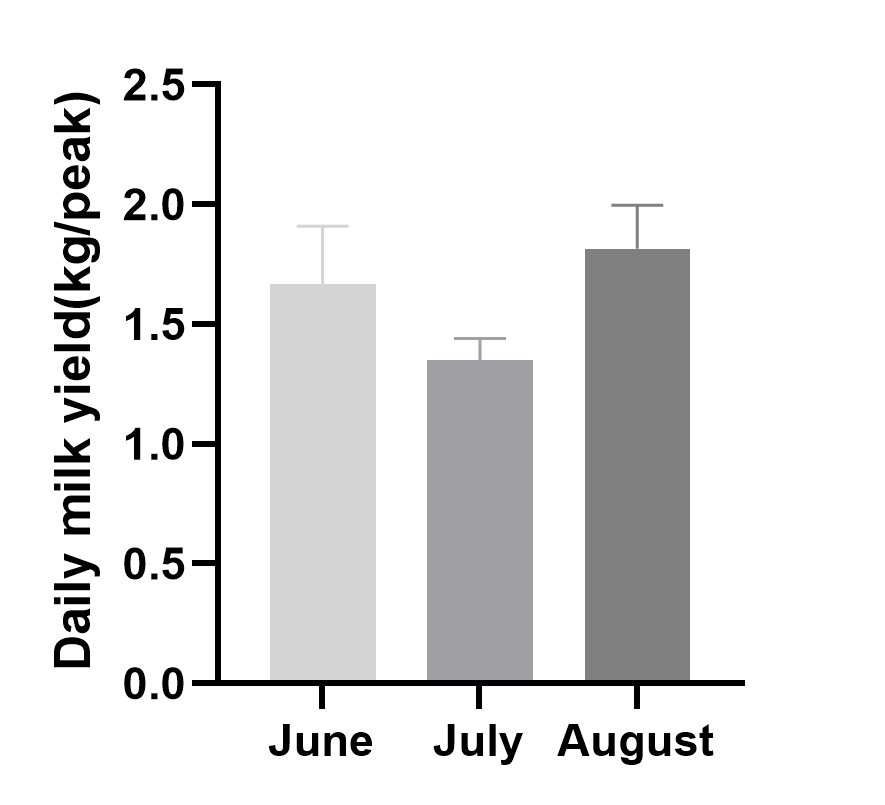


**Supplemental Figure 2**. Daily average milk yield of lactating camel from June to August.

| **Variable** | **Source of Variation** | **df** | **MS** | **F** | **P** |
| --- | --- | --- | --- | --- | --- |
| Camel milk yield | Time | 5 | 1.037 | 14.147 | ＜0.001 |
|  | Zinc Supplementation | 3 | 0.151 | 2.037 | 0.116 |
|  | Time*Zinc Supplementation | 15 | 0.044 | 0.592 | 0.872 |

**Supplemental Table 2.** Interaction effects of zinc supplementation dosage and experimental period on milk yield.

Data analysis utilized Interaction Effect Analysis.


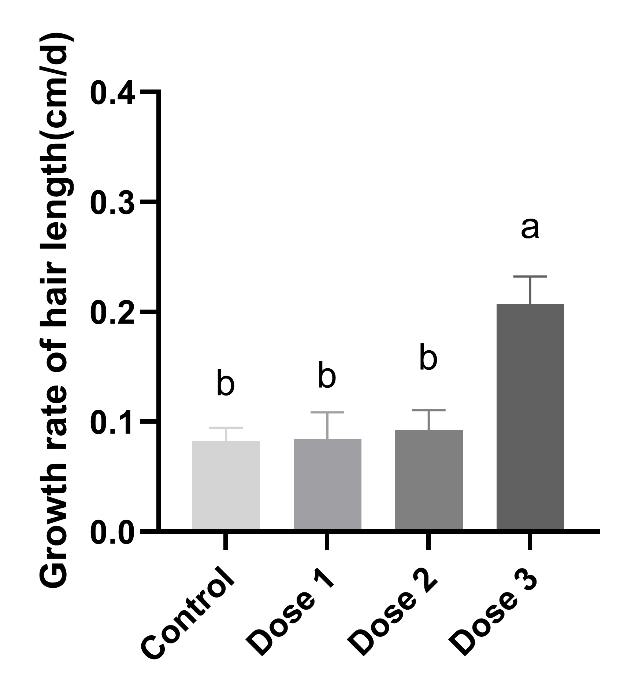

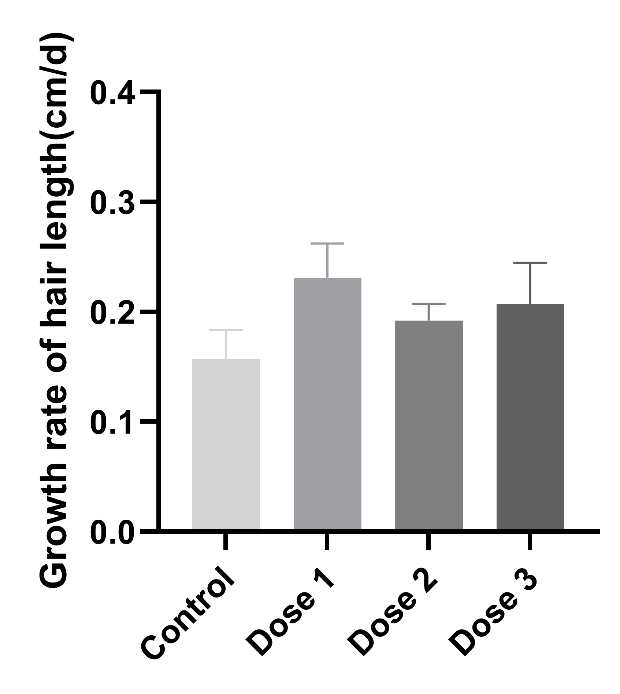

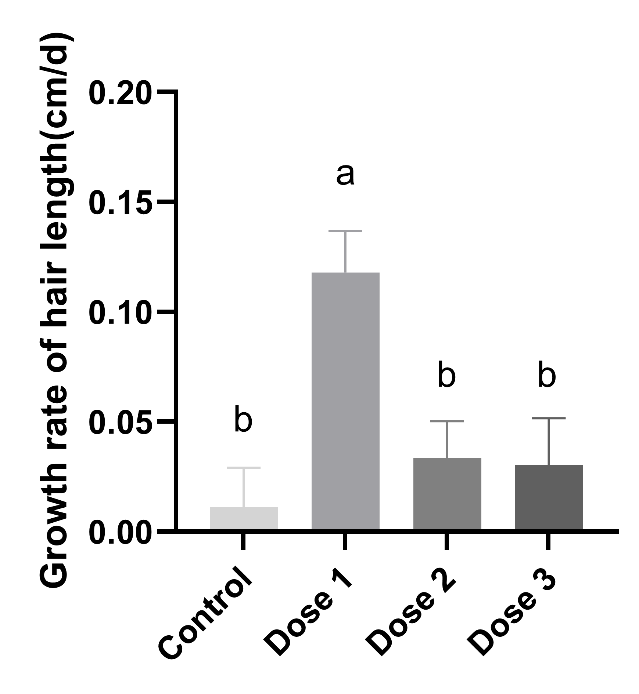

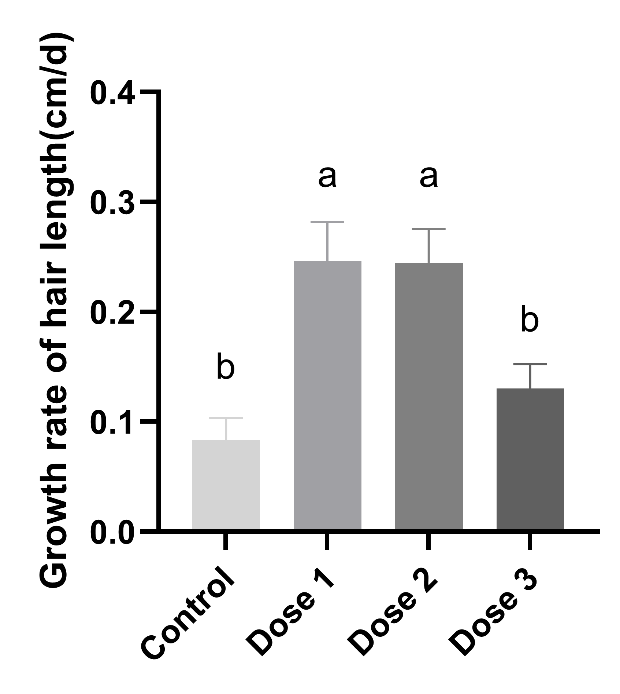


A

B

C

D

**Supplemental Figure 3**. Hair-growth rate in lactating camels and calves during the first two weeks and the subsequent two weeks. (A, B) Growth rate of neck hair in lactating camels during the first two weeks(A) and the subsequent two weeks(B). (C, D) Growth rate of neck hair in calves during the first two weeks(C) and the subsequent two weeks(D). ^a,b^ Different letters indicating significant differences among different groups (*p* < 0.05).
